# Un cas rare de pneumopéritoine spontané massif: à propos d’une Observation

**DOI:** 10.11604/pamj.2017.26.192.11554

**Published:** 2017-03-31

**Authors:** Mohamed Essarghini, Mohamed Tarchouli, Mohamed Elfahssi, Abdelmounim Aitali, Ahmed Bounaim

**Affiliations:** 1 Service de Chirurgie 1 de l’Hôpital Militaire d’Instruction Mohamed V, Rabat, Maroc

**Keywords:** Pneumopéritoine spontané, cœlioscopie, pneumopéritoine massif, Spontaneous pneumoperitoneum, laparoscopy, massive pneumoperitoneum

## Abstract

Le pneumopéritoine spontané est une affection rare caractérisée par la présence d’un épanchement d’air libre dans la cavité péritonéale en l’absence de toute cause évidente, nous rapportons une observation d’un pneumopéritoine massif de découverte scanographique dont l’exploration clinique, biologique, radiologique et cœlioscopique n’a aboutie a aucune étiologie décelable. Cette observation met le jour sur une entité clinique rarement vue dans notre pratique constituant un véritable piège diagnostic, sa connaissance éviterait des laparotomies blanches inutiles et surtout agressives pour nos patients. La cœlioscopie semble être un moyen chirurgical peu invasif pour les formes douteuses et permet un diagnostic visuel en éliminant une perforation d’organe creux.

## Abstract (en)

Spontaneous pneumoperitoneum is a rare disorder characterized by the presence of free air in the peritoneal cavity, in the absence of any obvious cause. We here report the case of a patient with massive pneumoperitoneum, detected by scan, in whom clinical, biological, radiological and laparoscopic examinations showed no detectable etiology. This case study describes a clinical entity rarely seen in our practice constituting a real diagnostic trap; accurate knowledge about the disease would avoid unnecessary and especially aggressive laparotomies revealing no signs of perforation. The laparoscopy seems to be a minimally invasive surgical procedure for doubtful cases allowing visual diagnosis by eliminating hollow organ perforation.

## Introduction

Le pneumopéritoine est un épanchement gazeux libre de la cavité péritonéale. Sa présence traduit dans 90% des cas une perforation du tractus gastro-intestinal [1]. En l’absence de toute perforation, on parle de pneumopéritoine spontané, une maladie rare et ne relevant pas toujours d’un traitement chirurgical [1, 2].

## Patient et observation

**Figure 1:**
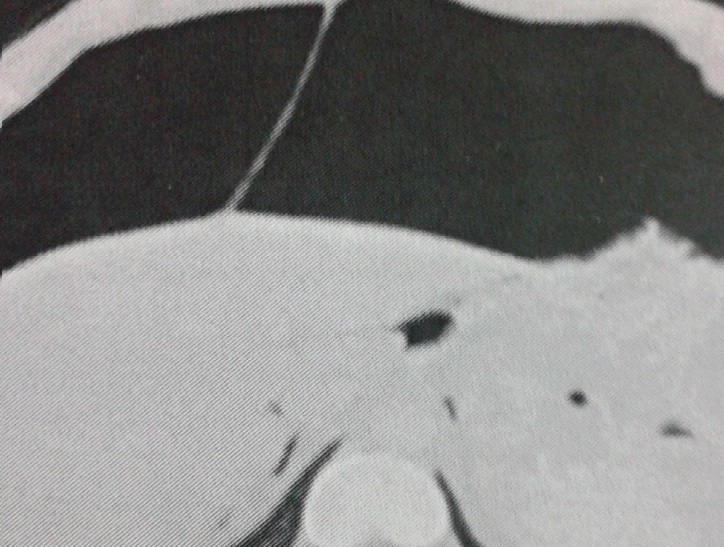
Aspect TDM d’un géant pneumopéritoine avec le foie refoulé en arrière

**Figure 2:**
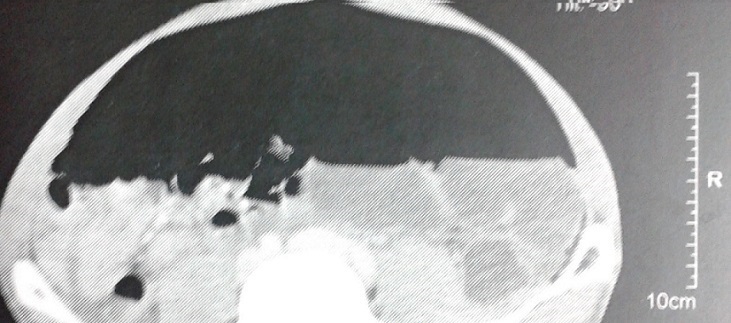
Aspect TDM du pneumopéritoine plaquant les anses contre le péritoine pariétal postérieur

Il s’agit d’un patient de 44 ans, sans antécédents qui a consulté aux urgences pour une douleur abdominale évoluant depuis trois jours, l’interrogatoire n’a pas révélé d’autres signes fonctionnels digestifs ou extradigestifs. L’examen clinique a trouvé un patient en bon état de santé générale, une tension artérielle normale à 12/8cmHg, un pouls à 80 cycle/min, une saturation d’O_2_ à 100% et une température à 37°C. L’examen abdominal a montré à l’inspection une distension abdominale généralisée, à la palpation une sensibilité diffuse et à la percussion un tympanisme. Les orifices herniaires entaient libres et le toucher rectal était normal. Un bilan biologique réalisé était sans particularité, le patient a été mis sous traitement symptomatique des troubles fonctionnels intestinaux. Mais vu l’absence d’amélioration clinique 24 heures après: la persistance de la douleur et l’exagération du météorisme, une tomodensitométrie abdominale a été réalisée. Elle a objectivé un pneumopéritoine géant en l’absence de collection, d’épanchement liquidien, ou d’une étiologie pouvant expliquer cet épanchement gazeux (Figure 1, Figure 2). Devant ce tableau clinique et la crainte persistante de méconnaitre et de laisser évoluer une pathologie sous jacente, on a décidé de faire une exploration coelioscopique, qui a éliminé une étiologie chirurgicale pouvant provoquer cet épanchement aerique, notamment un ulcère perforé, une appendicite, une diverticulose sigmoïdienne. Le diagnostic retenu pour notre patient était celui de pneumopéritoine spontané d’étiologie indéterminée.

## Discussion

Plusieurs mécanismes ont été décrits pour expliquer les étiologies du pneumopéritoine spontané dont les causes intrathoraciques comme les traumatismes, les pneumothorax, la ventilation mécanique ou la réanimation cardiopulmonaire. Chez la femme la perméabilité des trompes de Fallope peut justifier un passage d’air à travers le tractus génital suite a un rapport sexuel. L’insufflation au cours des explorations digestives endoscopique, peut aussi être incriminée comme cause iatrogène [3]. Devant un pneumopéritoine avec douleur abdominale les investigations cliniques, biologiques et l’imagerie s’orientent automatiquement vers une urgence chirurgicale abdominale et doivent rechercher en premier une perforation du tractus digestif, les étiologies les plus fréquemment rencontrées sont représentées par: la péritonite par perforation d’ulcère, une complication de MICI (maladie inflammatoire chronique de l’intestin), la diverticulose colique ou le cancer [3, 4]. La tomodensitométrie constitue l’imagerie de choix et peut orienter vers une étiologie abdominale évidente du pneumopéritoine [4-6] . Dans notre cas même si elle a confirmé la présence d’un pneumopéritoine massif, aucune orientation étiologique n’a été évoquée. Pour pousser l’enquête étiologique le recours à un « Look » Coelioscopique constitue un moyen chirurgical mini-invasif [4, 7]. Heureusement pour notre patient, cela a écarté l’éventualité d’une perforation d’organe creux mais aussi a permis une exsufflation a visée antalgique.

## Conclusion

En matière de pneumopéritoine spontané, la coelioscopie constitue un moyen chirurgical mini invasif a visée étiologique et thérapeutique mais le dogme de l’exploration chirurgicale à chaque fois qu’on constate un pneumopéritoine sur l’imagerie n’est pas toujours valide, en fin de compte, on opère des patients et non pas des images radiologiques.
